# Gyrophoric Acid, a Secondary Metabolite of Lichens, Exhibits Antidepressant and Anxiolytic Activity In Vivo in Wistar Rats

**DOI:** 10.3390/ijms252111840

**Published:** 2024-11-04

**Authors:** Nicol Urbanska, Martina Karasova, Zuzana Jendzelovska, Martin Majerník, Mariana Kolesarova, Dajana Kecsey, Rastislav Jendzelovsky, Peter Bohus, Terezia Kiskova

**Affiliations:** 1 Institute of Biology and Ecology, Faculty of Science, Pavol Jozef Safarik University in Kosice, 040 01 Kosice, Slovakia; 2 Small Animal Clinic, University of Veterinary Medicine and Pharmacy in Kosice, 041 81 Kosice, Slovakia; 3 Department of Pathology, Faculty of Medicine, Pavol Jozef Safarik University in Kosice, 040 01 Kosice, Slovakia

**Keywords:** gyrophoric acid, antioxidant, Wistar rats, depression-like behavior, neurogenesis, mature neurons, hippocampus, anxiolytic activity

## Abstract

Gyrophoric acid (GA) is a secondary metabolite of various lichens. It exhibits various biological activities in vitro, but only one study has been carried out in vivo. Because our previous study showed that GA stimulates neurogenesis in healthy rats, the current study aimed to explore the potential of GA during stress-induced depressive-like states in male Wistar rats. In the experiment, pregnant females were used. In the last week of pregnancy, females were subjected to restraint stress. After birth, progeny aged 60 days were stressed repeatedly. The males were divided into three groups: control animals (CTR; n = 10), males with a depression-like state (DEP; n = 10), and GA-treated animals (GA; n = 10). GA males were treated with GA (per os 10 mg/kg) daily for one month, starting from the 60th postnatal day. Our results indicate that GA acts as an antioxidant, as shown by a lowered ROS level in leukocytes (*p* < 0.01). Moreover, it prolonged the time spent in open arms in the elevated plus maze (*p* < 0.001). Concomitantly, the stimulation of proliferative activity in hippocampal regions was seen (hilus *p* < 0.01; subgranular zone *p* < 0.001) when compared with DEP males. Additionally, the number of mature neurons in the CA1 region of the hippocampus increased markedly (*p* < 0.01), indicating the role of GA in the maturation process of neurons. Thus, our study points to the potential anxiolytic/antidepressant activity of GA. However, future studies are needed in this complex area.

## 1. Introduction

There has been an “explosion” in anxiety disorders over the last decade worldwide. In the last year, anxiety disorders have affected 301 million people [1]. Their onset is usually in adolescence or early adulthood. The affected patients often develop further mental or somatic illnesses (sequential comorbidity) [2]. Moreover, mental health disorders represent an enormous cost to society and are related to economic outcomes [3]. 

Gyrophoric acid (GA) is a well-known lichen secondary metabolite which has been intensively studied in recent years. After its positive biological effects were revealed, the interest in it increased significantly. 

**Figure 1:**
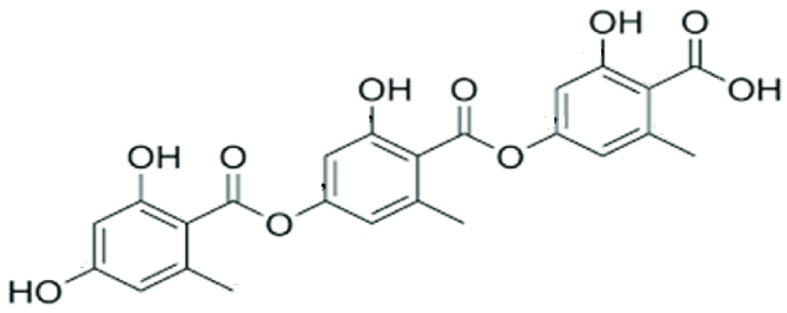
Chemical structure of gyrophoric acid modified according to [43].

GA is a tridepside from lichen of the genera *Umbilicaria* [4], *Austromelanelixia* [5], *Dactylina* [6,7], *Ochrolechia* [8], *Stereocaulon* [9], *Placopsis* [10], *Lecaimeria*, *Immersaria* [11], *Biatora* [12,13], *Parmelia* [14], *Psilolechia* [15], *Cryptothecia* [16], etc. GA is also produced by *Streptomyces* sp. IB 2014/I/ 78-8 [17], *Humicola* sp. FO-2942, and *Humicola* sp. FO-5962 [18]. Instead of a tridepside, GA was thought to be a didepside until this was disproved in 1925 and the structure was approved in 1933 [19]. The chemical structure of GA consists of three four-hydroxybenzoic acids connected by ester groups. Similarly, depsides consist of two four-hydroxybenzoic acids connected by ester groups, and tetradepsides consist of four four-hydroxybenzoic acids connected by ester groups (Figure 1) [20]. A study according to Singh (2022) identified the putative biosynthetic gene cluster of GA, the same cluster as that of hiascic and umbilicaric acid [21]. Among the main studied effects of GA are, for example, antimicrobial [22,23,24,25,26,27,28], UV-protective [29,30,31,32], antidiabetic [18,33,34], and anti-proliferative/cytostatic/cytotoxic activities [31,35,36,37,38,39], all in vitro. 

GA has been shown to possess antioxidant activities. Firstly, GA has a strong scavenging activity, as revealed using the Nishimiki method [40]. On the other hand, GA may serve as a prooxidant, as it is able to induce reactive oxygen species (ROS) production in HeLa cells, resulting in the launching of pro-apoptotic cascades [41].

Our previous research revealed that GA may change the behavior of healthy laboratory rats [42]. Because this is the only in vivo study, we decided to use the same dose of GA as used previously. As one lichen secondary metabolite, atranorin, acts as an anxiolytic/antidepressant compound, the aim of our study was to monitor the potential anxiolytic effects of GA at a dose of 10 mg/kg in Wistar rats. 

## 2. Results

### 2.1. Animal Behavior Analysis

**Figure 2:**
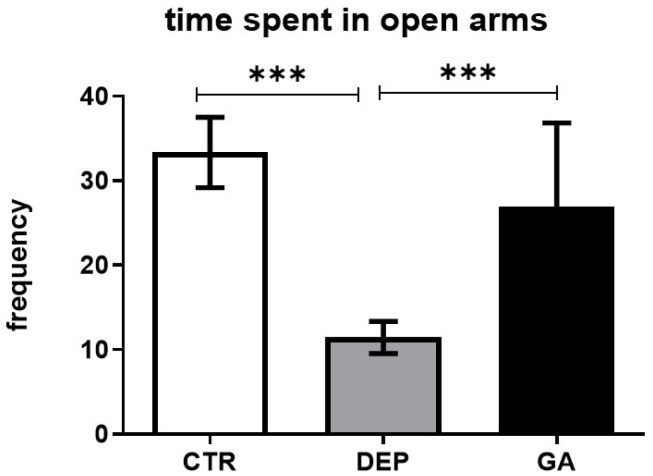
The time spent in the open arms in individual groups of control animals (CTR), animals with depression-like behavior (DEP), and gyrophoric acid-treated animals (GA) were evaluated using the elevated plus maze test. Values are presented as arithmetic mean ± SD. Significance is indicated by *** *p* ˂ 0.01.

Because the elevated plus maze (EPM) is a standard test for testing anxiety and depression-like behavior, we used it to measure the effects of GA during stress-induced depression. As shown in Figure 2, DEP animals spent markedly less time in the open arms of the apparatus. However, GA significantly increased the time spent in the open arms compared to the DEP group (*p* < 0.001). The other parameters in the tests were not affected (Table 1).

### 2.2. The Level of Hippocampal Neurogenesis 

**Figure 3:**
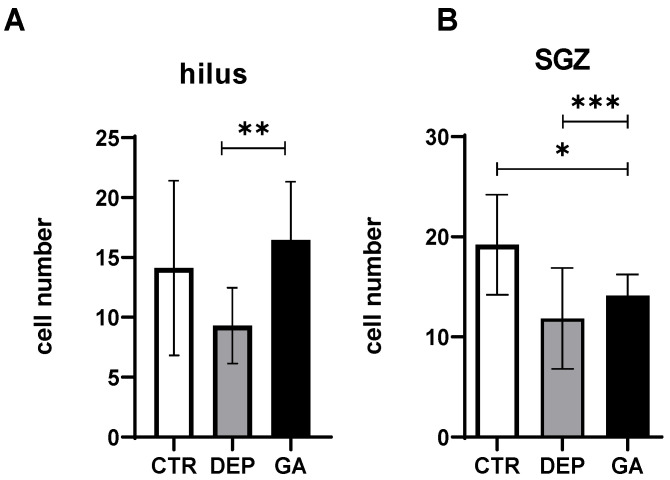
The number of proliferative Ki67-positive cells in the hilus (**A**) and SGZ (**B**) in individual groups of control animals (CTR), animals with depression-like behavior (DEP), and GA-treated animals (GA). Values are given as arithmetic mean ± SD. Significance is indicated by * *p* ˂ 0.05, ** *p* ˂ 0.01, and *** *p* ˂ 0.001.

A decrease in the number of Ki67 proliferating cells was observed in the DEP group compared to the CTR group in the hilus (Figure 3A) and subgranular zone (SGZ) (Figure 3B) of the hippocampus. The number of Ki67 proliferating cells was significantly increased in the GA group compared to the DEP group (*p* ˂ 0.01) in the hilus of the hippocampus (Figure 3A), and similarly, the increase in the number of Ki67 proliferating cells was significant (*p* ˂ 0.001) in the SGZ of the hippocampus (Figure 3B). 

### 2.3. Analysis of the Number of Neun-Positive Mature Neurons

**Figure 4:**
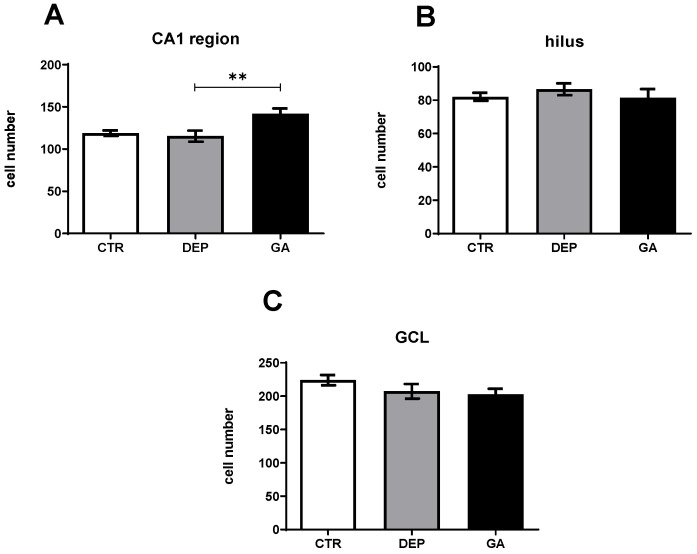
Number of NeuN-positive cells in the CA1 region (**A**), hilus (**B**), and granular cell layer (**C**) in individual groups of control animals (CTR), animals with depression-like behavior (DEP), and gyrophoric acid-treated animals (GA). Values are presented as arithmetic mean ± SD. Significance is indicated by ** *p* ˂ 0.01.

The number of NeuN-positive mature neurons in the DEP group stayed unchanged compared to the level of the neurons in the CTR group in the CA1 region and hilus and slightly decreased in granular cell layer (GCL). However, in the GA group, the number of NeuN-positive mature neurons was not increased compared to the DEP group in the hilus and in the GCL (Figure 4). In the GA group, the number of NeuN-positive mature neurons in the CA1 region was significantly increased (*p* ˂ 0.01) compared to the DEP group. 

### 2.4. Reactive Oxygen Species Levels in Leukocytes

**Figure 5:**
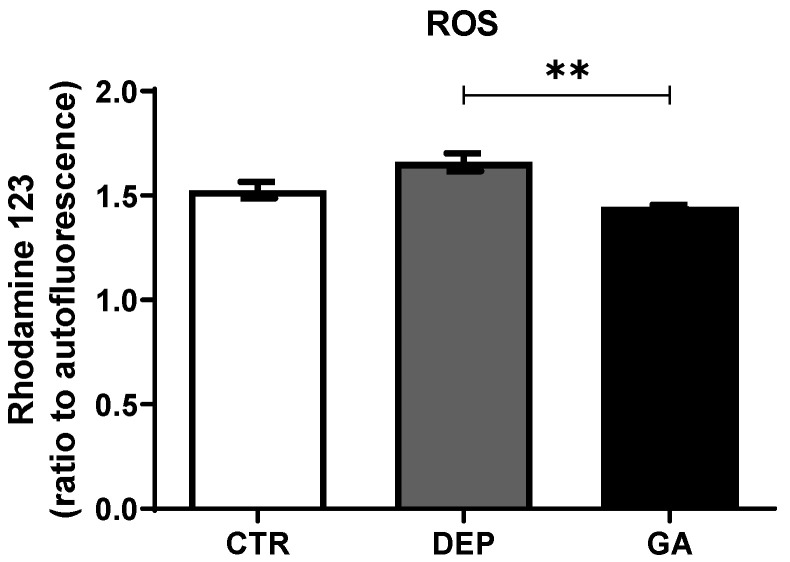
The level of reactive oxygen species in blood leukocytes in individual groups of control animals (CTR), animals with depression-like behavior (DEP), and gyrophoric acid-treated animals (GA). Values are presented as arithmetic mean ± SD. Significance is indicated by ** *p* ˂ 0.01.

GA significantly reduced ROS (*p* ˂ 0.01) in blood leukocytes compared to the DEP group. In the DEP group, there was a nonsignificant increase in ROS in blood leukocytes compared to the CTR group (Figure 5).

## 3. Discussion

Currently, the secondary metabolites of lichens are being tested for their medicinal potential mainly in vitro [7,36,44] but also in vivo [45,46]. Among the studied are the (mentioned above) antimicrobial, antibacterial, antifungal [22,23,24,25,26,27,28], UV-protective [29,30,31,32], anti-proliferative/cytostatic/cytotoxic [31,35,36,37,38,39], antioxidant [7,26,40,41,44,47,48,49], antidiabetic [18,33,34], and antiviral activities [50,51]—they have also shown activity against SARS-CoV-2 in vitro [52]. Positive effects are also shown by the combination of GA with usnic acid, which supports tissue regeneration [37]. Lichens can also be toxic [53], but because of the lack of in vivo reports, only one study has shown no hepatotoxicity after chronic GA consumption at a dose of 10 mg/kg [42]. 

### 3.1. Animal Behavior Analysis

After analyzing the behavior of the rats, our results showed increased time spent in the open arms in the GA group compared to the DEP group (Figure 2). Time spent in the open arms is the most important point of the test because it focuses on the anxiety of the animals. This is consistent with previously published data (the only in vivo study) showing that GA may increase not only the time spent in the open arms but also the rearing frequency and/or locomotor activity (as the number of center crossings) in healthy animals [42]. In our current experiment, other forms of behavior were not changed in the EPM (Table 1). This could be influenced by the unpredictability of the behavior of the animals. 

Another lichen secondary metabolite, atranorin, has been studied in relation to depression [46]. This study demonstrated the antidepressant effect of atranorin at a dose of 10 mg/kg by increasing the time spent in the open arms in the EPM compared to the depressed group of males, which is similar to the results of the current study (Figure 1). In another study, the time spent in the open arms in the EPM was increased after the administration of the lichen metabolite atranorin to healthy animals at a concentration of 10 mg/kg compared to the control untreated group of animals of both sexes [45]. The time spent in the open arms of the EPM is one of the parameters used in the assessment of anxiety [54]. However, due to the high comorbidity of anxiety and depression, it is often not possible to separate their symptoms [55]. The EPM is highly sensitive to pharmacological and behavioral treatments [54]. Anxious and depressive behavior has been confirmed in animals and humans [56,57]. Our results show a decrease in exploratory behavior, expressed by the time spent in the open arms in the EPM in DEP animals. In a study in depressed patients, a reduction in exploratory behavior was also observed [58]. In the studies, the metabolites of lichens were administered orally [42,45,46]. One in vitro study found that the gastrointestinal absorption of GA, salazinic acid, and fumarprotocetraric acid was low, and GA had a low bioavailability score (predicts the fraction of an orally administered compound that reaches systemic circulation), which was 0.11. GA fulfills Lipinski’s five rules. In contrast, gastrointestinal absorption was high for the compounds usnic acid, cryptostictinolide, variolaric acid, and norstictic acid [52]. The di-methylated analogue of GA—tenuiorin—showed the prevention of the neurodegenerative disease Alzheimer’s disease by inhibiting tau protein aggregation at a concentration of 75 and 100 μM [59]. In a model of Alzheimer’s disease in vivo (exposed to peptide Aβ1-42—400 pmol/mouse—intracerebroventricular), following the oral administration of 25, 50, and 100 mg/kg of different enantiomers (R)-(+)-UA and (S)-(- )-UA of usnic acid, after 24 h, cognitive deficits, the consequences of inflammation, and the action of lipid hydroperoxides were improved. The enantiomers of usnic acid improved memory and learning in mice, reduced the activity of lipid hydroperoxides and myeloperoxidase in the cortex and hippocampus, and reduced the levels of IL-1β in the hippocampus [60]. These studies together with our results indicate that depsides, including GA, may have neurotrophic, neuroprotective, anxiolytic, or antidepressant effects.

### 3.2. Analysis of the Number of Proliferative Ki67 Cells and Mature Neun-Positive Neurons

Exploratory behavior is dependent, among other brain structures, on the medial temporal lobe and hippocampus, which links spatial conceptions from its function with general memory-based approaches to hippocampal function [61,62]. The neurogenic hypothesis establishes a connection between depression and neurogenesis. The consequence of depression, according to the hypothesis, is a reduction in neurogenesis, which can be restored by antidepressants [63]. A decrease in hippocampal neurogenesis, memory impairment, and an increase in the activity of the hypothalamus–pituitary gland also occur after the exposure of rats and monkeys to prenatal stress, which results in an increase in anxiety and depression during their lifetime [64,65]. In our study, the number of proliferative Ki67-positive cells in the hilus of the hippocampus was evaluated, as this is where the precursors of proliferating cells are located [66,67]. In the hilus, the number of proliferative Ki67-positive cells was reduced in the DEP group compared to the CTR group (Figure 3). Thus, the repeated prenatal and postnatal stress was sufficient to induce anxiety- and depression-like states in this study. Increased levels of glucocorticoids could reduce proliferative activity after chronic stress [68,69]. During mitosis, neural stem/progenitor cells (NPCs) do not express glucocorticoids (GRs) and mineralocorticoid receptors (MRs). GRs are expressed 24 h and MRs 2 weeks (after migration to the GCL and cell division) after mitosis [70]. Increased levels of GRs may be a consequences of increased glutamate levels, and subsequently, glutamate may reduce cell proliferation [71,72,73]. Concomitantly with the changed behavior of the animals after GA consumption, our results demonstrate that GA also increased the level of neurogenesis in the hippocampus (Figure 3). Moreover, GA was also able to increase the number of mature neurons in the CA1 region, which decreased after depression, as shown in the DEP group (Figure 4). The CA1 area is sensitive to metabolic stress in various diseases such as ischemia, hypoglycemic encephalopathy, limbic encephalitis, multiple sclerosis, epilepsy, and transient global amnesia. The mechanism of sensitivity is poorly understood and includes oxidative stress and genetically determined, glutamate-dependent, and calcium-mediated mechanisms of neuronal excitotoxicity [74].

As we know, mature neurons can differentiate from proliferative cells in the hippocampus [75]. Antidepressants and exercise can accelerate the maturation of neurons [76]. Increased NeuN-positive cells in the GCL and CA1 area of the hippocampus were also found in our previous study after the application of atranorin [46]. A neuroprotective, neurotrophic, and proneurogenic effect was also confirmed in other lichen metabolites, such as with perlatolic acid, which induces a higher level of neutrophic factors than BDNF and NGF, resulting in neurite outgrowth in vitro at 0.5 μM. Other depsides, such as atranorin and physodic acid, have shown potential neuroprotective and neurotrophic activity. BDNF and NGF production activity is related to the increased histone H3 and H4 acetylation in a mouse neuroblastoma cell line (Neuro2A). Perlatolic acid strongly inhibits acetylcholinesterase (AChE) in vitro [77]. Usnic acid also interacted with AChE in a mouse model of Parkinson’s disease [60]. Similarly, atranorin was proneurogenic and neurotrophic at a concentration of 5 μM, resulting in neurite outgrowth in Neuro 2A cells. Atranorin stimulated the expression of neurotrophic genes for BDNF and NGF [78]. BDNF deficiency causes neuroinflammation, which can lead to symptoms of major depressive disorder [79]. Through a mechanism dependent on the Ras/ERK pathway, BDNF increases synaptic spine density. Synaptic plasticity is impaired in depression by the dysfunction of or reduction in BDNF levels, reduction in excitatory neurons, and glutamate [80]. In addition, BDNF also plays an important role in neurogenesis and neuronal development. It stimulates NPCs [81,82,83]. NPCs give rise to glial and neuronal cells in the CNS [84]. BDNF stimulates hippocampal neurogenesis after intrahippocampal injection and peripheral injection [83,85,86]. The mechanism of neurogenesis was confirmed in the dentate gyrus via BDNF-LTP (long-term potentiation). The induction of BDNF-LTP and the proneurogenic effect is blocked by the inhibition of activity-regulated cytoskeleton-associated protein (Arc) translation [87]. The mechanisms remain to be investigated, but acute BDNF injection in adult rodents is associated with the induction of Arc-dependent LTP [88]. 

### 3.3. Reactive Oxygen Species Levels in Leukocytes

As the pathophysiology of neurodegenerative diseases, including depression, involves oxidative stress [7], we decided to monitor the levels of ROS in circulating blood. Indeed, a slight increase in ROS level in leukocytes was revealed in DEP animals. However, GA decreased their level to the control level (Figure 5). 

Previously, GA significantly reduced ROS in human melanoma A375 cells at a concentration of 50 μM for 72 h [89]. Our results indicate an antioxidant effect of GA. The depsidone chemical structure of GA with hydroxyl groups that interact with enzymatic active sites and aromatic rings is responsible for its antioxidant activity and scavenging of free radicals [20,90,91,92]. The redox nature of lichens depends not only on their structure but also on the system in which they are found. It is considered that it may also depend on the concentration of the given metabolite, but there is no direct evidence for this yet [10]. Lichen metabolites induced the expression of antioxidant enzymes [93,94,95], playing a key role in various diseases [96,97]. In silico, GA demonstrated a strong affinity for the GPCR receptor [98], which links various cellular responses to neurotransmitters and hormones, e.g., taste, vision, and olfaction [99]. A recent study noted that the depsidone structure of GA strongly scavenges hydroxyl and superoxide anions in a polar environment but weakly scavenges peroxyl radical scavengers [100]. In vitro, the antioxidant property of GA was recorded using the 2,2-diphenyl-1-picrylhydrazyl (DPPH) radical scavenging activity method at a GA concentration of 0.1–5 mg/L [22], and another in vitro study confirmed the DPPH radical scavenging activity and also that GA (62.5–1000 μg/mL) achieved the highest values in superoxide anion scavenging IC50 compared to ascorbic acid (vitamin C) [101]. However, a similar study compared to the previous study points to a lower antioxidant effect of GA in DPPH radical scavenging activity and in the scavenging of superoxide anions compared to ascorbic acid. The antioxidant effect was 4–13%, and the uptake of superoxide anions was 51–63%. The percentages demonstrate the marginal effect of other secondary metabolites of lichens as well. Separate efficiency percentages for GA are not specified in the study [40]. GA in reduction power (the value of an antioxidant in donating an electron to a free radical for its neuralization, the mechanism of phenolic antioxidants) reached the highest values (at a concentration of 62.5–1000 μg/mL) among the tested samples; it exceeded ascorbic acid [102,103]. Another depside, atranorin, showed an antioxidant effect dependent on free radicals such as superoxide, hydroxyl radicals, H_2_O_2_, and NO in in vitro studies at a concentration of 100 μg/mL [104,105] and scavenged superoxide anion at an IC50 concentration of 169.65 μg/mL [106].

It remains unclear how GA stimulates the process of neurogenesis in the brain. Some natural substances such as resveratrol, quercetin, and lovastatin pass into the CNS by passive transport [107,108], but some secondary metabolites of lichen such as phyzodic acid and atranorin can also pass through the blood–brain barrier (BBB) [109]. Evernic acid and physodic acid pass through the BBB as a pure substance but also in extract form. However, salazinic acid is not able to pass through the BBB as a pure substance or in the form of an extract, which may indicate a difference in the chemical structure of the bioactive substances [110,111]. In rats, it has been shown that GA passes through the BBB in a low manner [42]. However, another in vitro study found out that GA does not [52]. Thus, more studies are needed to clarify this question. Concomitantly with our previous study, showing the potential of GA passing the BBB [42], in the chemical structure of some lichen secondary metabolites, there are alkyl chains that can improve the affinity to receptors, increase the lipophilic properties of the molecules, and thus improve the passage through the BBB [78]. However, our findings of the GA stimulation of neurogenesis and alteration of spatiotemporal regulation indicate that GA may influence the brain structures and concomitantly also behavior. 

Our results describe the anxiolytic/antidepressant effect of GA in a model of restraint- stress-induced depression. They provide the first evidence of the effects of GA during depression. Based on our observations, the lichen secondary metabolite GA has potential in the treatment of depression or other neurodegenerative disorders and should be considered in future studies.

## 4. Materials and Methods

### 4.1. Experimental Design and Laboratory Animals

In this study, a parental generation including 2 Wistar males and 4 Wistar females was used (Department of Toxicology and Laboratory Animal Breeding ÚEFT SAV, Dobrá Voda, Slovakia). The animals were housed in standard vivarium conditions, with a room temperature of 21–24 °C, a relative humidity of 50–65%, and a 12/12 light/dark regimen. The animals had access to tap water and pelleted food (Velaz, Únetice, Prague, Czech Republic) ad libitum, following EU regulations and guidance on animal feeding. Parental females were mated with parental males, and male progeny were selected for subsequent experiments. The animals were managed according to the guidelines set forth in Law No. 377 and 436/2012 of the Slovak Republic for the Care and Use of Laboratory Animals, which were approved by the State Veterinary and Food Administration of the Slovak Republic (Approval Number: Ro-2866/16-221, Ro-2219/19-221/3).

At the end of the experiment, transcardial lavage under deep anesthesia (400 mg/kg of chloral hydrate, administered intraperitoneally) was used to euthanize the laboratory animals. Perfusion through the left ventricle began with a saline wash of the blood vessels, followed by perfusion with a 4% freshly prepared paraformaldehyde solution. Subsequently, the brains were removed, weighed, and post-fixed in the same fixative for 24 h, after which they were transferred to a 30% sucrose solution for cryoprotection. Then, 33 μm coronal sections of the brain were cut using a cryostat at the level of the bregma −3.3 ± 0.2 mm.

### 4.2. Induction of Depression-like Behavior in Animals

During the experiment, pregnant females were divided into two groups: the first group was a control group of healthy females, and the second group was subjected to restraint stress. The second group of pregnant females was subjected to restraint stress using immobilization boxes in the last week of pregnancy for three consecutive days, three times a day for 45 min according to a scheme that was previously used by other authors [46,112]. The progeny of the males were kept with their mothers for 30 days. Subsequently, the male progeny were divided into three groups: CTR—progeny of control mothers (n = 10); DEP—progeny of mothers subjected to restraint stress (n = 10); GA—progeny of mothers subjected to restraint stress and which were treated with GA extract (n = 10). GA at a dose of 10 mg/kg was perorally administered daily for one month, starting after postnatal stress. The secondary metabolite GA was provided by doc. RNDr. Michal Goga, PhD (isolated in the laboratory of the Department of Botany, UPJŠ). Briefly, *Umbilicaria hirsute* (Sw. Ex Westr.) was collected from extrusive igneous volcanic rocks Sninský kameň (48°55′46″ N 22°11′23″ E) in Vihorlat Mountains (Prešov, Slovakia). The lichen thalli were quickly washed with distilled water and carefully removed from the rock surface. Extraction of lichen material was performed using acetone as described previously [41]. 

### 4.3. Elevated Plus Maze Test 

The EPM was used to observe depression-like behavior and determine the anxiolytic effect of GA. The apparatus consisted of a cross-shaped maze with two open and two closed arms, each 80 cm in length, and a central square measuring 15 × 15 cm. The maze was positioned 75 cm above the ground. The level of depression and anxiety was assessed based on the time spent in the open arms of the maze and the frequency of defecation. Exploratory activity was measured by counting the number of rearing behaviors, while comfort behaviors were quantified by counting the number of washing acts. Locomotor activity was recorded by noting the number of passes through the center of the maze. By default, each animal was tested for 5 min.

### 4.4. Collection of Blood

At the end of the experiment, blood samples were obtained from the vena saphena of each animal. The blood was collected into microtubes containing an appropriate volume of heparin. 

### 4.5. Assessment of Reactive Oxygen Species in Leukocytes

Blood samples (100 µL) were collected from rats in heparin-treated tubes. Using red blood cell lysis buffer (150 mM NH4CI, 10 mM KHC03, 0.1 mM EDTA, pH 7.4), red blood cells were lysed for 3 min. Samples were centrifuged at 200× *g* for 6 min, and the pellet was washed with 1 mM PBS-EDTA. Subsequently, each sample was divided in half; one half was stained, and the other half was used as an unstained autofluorescent control. Whole blood samples were stained with 10 μM 3 dihydrorhodamine-123 (DHR 123, Fluka, Buchs, Switzerland) for 20 min at room temperature in the dark, following previously established protocols [35]. The samples were evaluated by a BD FACSCalibur (BD) flow cytometer (Becton Dickinson, San Jose, CA, USA) with a 488 nm argon-ion excitation laser. Debris was eliminated by forward scattering and side scattering (FSC × SSC). Fluorescence was detected using a 530/30 bandpass filter (FL-1) and quantified using FlowJo software (Tree Star, Inc., Ashland, OR, USA). The level of ROS in leukocytes was expressed as the ratio of the median fluorescence of DHR 123 to the autofluorescence of unstained samples. 

### 4.6. Immunohistochemical Staining of Ki-67-Positive Cells in the Hippocampus 

Ki67 is a reliable proliferation marker of neoplastic and/or normal cells, according to Gerdes et al. (1984) [113] and Kee et al. (2002) [114]. Immunohistochemical staining was performed on free-floating coronal sections. Briefly, sections were incubated in blocking buffer and then incubated at 4°C overnight with primary antibody (Ki-67 (D3B5) rabbit mAb, #12202; dilution 1:400; Cell Signaling Technology, Danvers, MA, USA). The incubation of sections was performed with goat anti-rabbit IgG secondary antibody (Vector Laboratories, 1:200) for 1 h. The incubation was performed with a dilution of 1:400. Subsequently, the incubation of sections in avidin–biotin–peroxidase complex (Vector Laboratories, Burlingame, CA, Vestastain ABC kit) was performed for 1 h at room temperature, and DAB (12 mmol L^−1^ concentration in PBS with 0.003% H_2_O_2_, Sigma-Aldrich, St Louis, MO) application lasted 10 min. After the dehydration protocol, they were sprinkled with Permount (Fischer Scientific, Pittsburg, PA, USA).

### 4.7. Immunohistochemical Staining of Neun-Positive Mature Neurons in the Hippocampus

Immunohistochemical staining was performed on free-floating coronal sections. Briefly, sections were incubated with anti-NeuN antibody (MAB377, 1:500; Millipore, Bedford, MA, USA) in 0.1 mol/L PBS (pH 7.4) with 0.3% Triton at 4 °C overnight. Sections were washed with 0.1 mol/ L PBS (pH 7.4) with 0.2% Triton, and then, a secondary anti-mouse IgG antibody (BA-2000, 1:200) was applied at room temperature for 90 min. The application of ABC Elite (Vector Laboratories, Burlingame, CA, USA) for 90 min was performed after washing the sections, followed by rinsing the sections with PBS and applying DAB (0.1 mol/L Tris, 0.04% DAB, 0.033% H_2_O_2_); the reaction was stopped with phosphate buffer. This was followed by the dehydration of the sections, followed by the application of Permount (Fischer Scientific, Pittsburg, PA, USA) and the cover-slipping of the sections for analysis [115].

### 4.8. Assessment of Cell Number

Photomicrographs of brain sections were obtained using a light microscope (Leica DM2500) and analyzed using ImageJ (ImageJ, Bethesda, MD, USA). The quantification of cell number was performed on every sixth cryostat hippocampal section. The number of NeuN-positive cells was counted in different areas of the hippocampus: in the CA1 area, its mid-linear part (at 200× magnification), in the total hilus area (at 100× magnification), and in the GCL area (at 200-fold magnification), as previously described [116]. The absolute number of cells for a selected 400 μm section of each examined section represents data on NeuN-positive cells in the CA1 and GCL regions. The number of Ki67-positive cells was counted in the whole region of the hilus and SGZ DG at 100× magnification.

### 4.9. Statistical Evaluation

GraphPad Prism 8.0.1 (GraphPad Software, Inc., San Diego, CA, USA) was used for all statistical analyses. All data were assessed for normal distribution, and appropriate statistical tests were applied. The results are presented as mean ± standard deviation (SD).

## 5. Conclusions

The antioxidant and anxiolytic effect of GA simultaneously with the behavior and neurogenesis of the hippocampus points to its potential importance in the research of mental diseases. The positive effect of GA on behavior during depression-like behavior and the increased neurogenesis of the hippocampus support one of the many hypotheses about the effect of anxiolytics and antidepressants—the neurogenic hypothesis. The reduced ROS level in the blood after GA application supports the claim of the involvement of oxidative stress in the anxiolytic effect of GA during depression-like behavior. GA should be included in future research in the development of anxiolytics and antidepressants.

## Figures and Tables

**Table 1:** The parameters of elevated plus maze test (frequency) in individual groups of control animals (CTR), animals with depression-like behavior (DEP), and gyrophoric acid-treated animals (GA).

	CTR	DEP	GA
**Center crossings**	3.24 ± 3.05	3.57 ± 3.00	3.32 ± 2.54
**Washing**	4.76 ± 2.72	4.75 ± 2.68	4.68 ± 2.83
**Rearing**	13.80 ± 6.30	14.32 ± 5.69	13.32 ± 6.53
**Defecation**	0.22 ± 0.99	0.20 ± 0.95	0.21 ± 0.94

## Data Availability

Data can be shared after contacting the corresponding author.

## References

[1] Javaid S.F., Hashim I.J., Hashim M.J., Stip E., Samad M.A., Ahbabi A.A. (2023). Epidemiology of anxiety disorders: Global burden and sociodemographic associations. Middle East Curr. Psychiatry.

[2] Ströhle A., Gensichen J., Domschke K. (2018). The diagnosis and treatment of anxiety disorders. Dtsch. Ärzteblatt Int..

[3] Tham W.W., Sojli E., Bryant R., McAleer M. (2021). Common mental disorders and economic uncertainty: Evidence from the COVID-19 pandemic in the US. PLoS ONE.

[4] Norouzi H., Sohrabi M., Yousefi M., Boustie J. (2023). Tridepsides as potential bioactives: A review on their chemistry and the global distribution of their lichenic and non-lichenic natural sources. Front. Fungal Biol..

[5] Divakar P.K., Crespo A., Kraichak E., Leavitt S.D., Singh G., Schmitt I., Lumbsch H.T. (2017). Using a temporal phylogenetic method to harmonize family-and genus-level classification in the largest clade of lichen-forming fungi. Fungal Divers..

[6] Ureña-Vacas I., González-Burgos E., De Vita S., Divakar P.K., Bifulco G., Gomez-Serranillos M.P. (2022). Phytochemical Characterization and Pharmacological Properties of Lichen Extracts from Cetrarioid Clade by Multivariate Analysis and Molecular Docking. Evid.-Based Complement. Altern. Med..

[7] Ureña-Vacas I., González-Burgos E., Divakar P.K., Gómez-Serranillos M.P. (2022). Lichen extracts from Cetrarioid clade provide neuroprotection against hydrogen peroxide-induced oxidative stress. Molecules.

[8] Rather L.J., Mir S.S., Ganie S.A., Islam S.-U., Li Q. (2022). Research progress, challenges, and perspectives in microbial pigment production for industrial applications—A review. Dye. Pigment..

[9] Torres J.M., Torres V.d.O., Rodrigues A.S., Gianini A.S., Micheletti A.C., Honda N.K., Spielmann A.A., Lorenz A.P. (2023). Lineages of the lichen-forming fungus Stereocaulon alpinum and their photobionts in southern South America and maritime Antarctica. Polar Biol..

[10] Poulsen-Silva E., Gordillo-Fuenzalida F., Atala C., Moreno A.A., Otero M.C. (2023). Bioactive lichen secondary metabolites and their presence in species from Chile. Metabolites.

[11] Xie C.-M., Wang L.-S., Zhao Z.-T., Zhang Y.-Y., Wang X.-Y., Zhang L.-L. (2022). Revision of Immersaria and a new lecanorine genus in Lecideaceae (lichenised Ascomycota, Lecanoromycetes). MycoKeys.

[12] Lendemer J.C. (2023). Studies in Lichens and Lichenicolous Fungi–No. 23: Notes on Appalachian taxa including newly reported disjunctions and multiple species new to North America. Opusc. Philolichenum.

[13] Printzen C., Holien H., Kantelinen A., Myllys L., Ratschow F., Stepanchikova I., Weber L., Timdal E. (2023). DNA barcoding indicates the presence of unrecognized species and phylogenetic diversity within the Biatora vernalis-and B. meiocarpa-groups. Plant Fungal Syst..

[14] Sharma B., Fatima S., Rajeshkumar K.C., Škaloud P., Divakar P.K., Gaikwad S., Ansil P.A., Mohan A.S., Sequeira S.Y. (2023). Molecular studies of Flavopunctelia and Punctelia species and their Trebouxia photobiont from the Himalayas, India. Mycotaxon.

[15] Palice Z., Svoboda S., Vondrák J. (2023). Hidden in the dark under umbrellas: Two new Psilolechia species (lichenized Ascomycota, Lecanorales) described from the Czech Republic. Plant Fungal Syst..

[16] Aptroot A., da Silva Cáceres M.E., dos Santos L.A. (2024). The taxonomy of sterile Arthoniaceae from Brazil: White crusts on overhanging tropical trees can be named. Lichenologist.

[17] Axenov-Gibanov D.V., Voytsekhovskaya I.V., Tokovenko B.T., Protasov E.S., Gamaiunov S.V., Rebets Y.V., Luzhetskyy A.N., Timofeyev M.A. (2016). Actinobacteria isolated from an underground lake and moonmilk speleothem from the biggest conglomeratic karstic cave in Siberia as sources of novel biologically active compounds. PLoS ONE.

[18] Inokoshi J., Takagi Y., Uchida R., Masuma R., Ōmura S., Tomoda H. (2010). Production of a new type of amidepsine with a sugar moiety by static fermentation of Humicola sp. FO-2942. J. Antibiot..

[19] Canter F.W., Robertson A., Waters R.B. (1933). 123. Lichen acids. Part V. A synthesis of methyl O-tetramethylgyrophorate. J. Chem. Soc. (Resumed).

[20] Manojlovic N.T., Vasiljevic P.J., Maskovic P.Z., Juskovic M., Bogdanovic-Dusanovic G. (2012). Chemical composition, antioxidant, and antimicrobial activities of lichen Umbilicaria cylindrica (L.) Delise (Umbilicariaceae). Evid.-Based Complement. Altern. Med..

[21] Singh G., Calchera A., Merges D., Valim H., Otte J., Schmitt I., Dal Grande F. (2022). A candidate gene cluster for the bioactive natural product gyrophoric acid in lichen-forming fungi. Microbiol. Spectr..

[22] Buçukoglu T.Z., Albayrak S., Halici M.G., Tay T. (2013). Antimicrobial and Antioxidant Activities of Extracts and Lichen Acids Obtained from Some U mbilicaria Species from C entral A natolia, T urkey. J. Food Process. Preserv..

[23] Candan M., Yılmaz M., Tay T., Kıvança M., Türk H. (2006). Antimicrobial activity of extracts of the lichen Xanthoparmelia pokornyi and its gyrophoric and stenosporic acid constituents. Z. Für Naturforschung C.

[24] Furmanek Ł., Seaward M.R. (2023). Anti-yeast potential of lichen-extracted substances—An analytical review. S. Afr. J. Bot..

[25] Jacobs B.L., Van Praag H., Gage F. (2000). Adult brain neurogenesis and psychiatry: A novel theory of depression. Mol. Psychiatry.

[26] Kosanić M., Ranković B. (2011). Antioxidant and antimicrobial properties of some lichens and their constituents. J. Med. Food.

[27] Madrigal J., Caso J., De Cristobal J., Cardenas A., Leza J., Lizasoain I., Lorenzo P., Moro M. (2003). Effect of subacute and chronic immobilisation stress on the outcome of permanent focal cerebral ischaemia in rats. Brain Res..

[28] Ranković B., Mišić M., Sukdolak S. (2008). The antimicrobial activity of substances derived from the lichens Physcia aipolia, Umbilicaria polyphylla, Parmelia caperata and Hypogymnia physodes. World J. Microbiol. Biotechnol..

[29] Gupta A., Singh A.P., Sahu N., Jaiswal J., Kumari N., Singh P.R., Sinha R.P. (2023). Bioprospecting and Evolutionary Significance of Photoprotectors in Non-flowering Lower Plants. Photoprotective Green Pharmacology: Challenges, Sources and Future Applications.

[30] Lohézic-Le Dévéhat F., Legouin B., Couteau C., Boustie J., Coiffard L. (2013). Lichenic extracts and metabolites as UV filters. J. Photochem. Photobiol. B Biol..

[31] Russo A., Piovano M., Lombardo L., Garbarino J., Cardile V. (2008). Lichen metabolites prevent UV light and nitric oxide-mediated plasmid DNA damage and induce apoptosis in human melanoma cells. Life Sci..

[32] Varol M., Türk A., Candan M., Tay T., Koparal A.T. (2016). Photoprotective activity of vulpinic and gyrophoric acids toward ultraviolet B-induced damage in human keratinocytes. Phytother. Res..

[33] Choudhary M.I., Ali M., Wahab A.-T., Khan A., Rasheed S., Shyaula S.L., Rahman A.-U. (2011). New antiglycation and enzyme inhibitors from Parmotrema cooperi. Sci. China Chem..

[34] Thadhani V.M., Karunaratne V. (2017). Potential of lichen compounds as antidiabetic agents with antioxidative properties: A review. Oxidative Med. Cell. Longev..

[35] Bačkorová M., Bačkor M., Mikeš J., Jendželovský R., Fedoročko P. (2011). Variable responses of different human cancer cells to the lichen compounds parietin, atranorin, usnic acid and gyrophoric acid. Toxicol. Vitr..

[36] Bačkorová M., Jendželovský R., Kello M., Bačkor M., Mikeš J., Fedoročko P. (2012). Lichen secondary metabolites are responsible for induction of apoptosis in HT-29 and A2780 human cancer cell lines. Toxicol. Vitr..

[37] Burlando B., Ranzato E., Volante A., Appendino G., Pollastro F., Verotta L. (2009). Antiproliferative effects on tumour cells and promotion of keratinocyte wound healing by different lichen compounds. Planta Medica.

[38] Castañeta G., Villagomez R., Salamanca E., Canaviri-Paz P., Bravo J.A., Vila J.L., Bárcenas-Pérez D., Cheel J., Sepúlveda B., Giménez A. (2023). Microwave-Assisted Semisynthesis and Leishmanicidal Activity of Some Phenolic Constituents from Lichens. Separations.

[39] Kumar KC S., Müller K. (1999). Lichen metabolites. 2. Antiproliferative and cytotoxic activity of gyrophoric, usnic, and diffractaic acid on human keratinocyte growth. J. Nat. Prod..

[40] Elečko J., Vilková M., Frenák R., Routray D., Ručová D., Bačkor M., Goga M. (2022). A comparative study of isolated secondary metabolites from lichens and their antioxidative properties. Plants.

[41] Goga M., Kello M., Vilkova M., Petrova K., Backor M., Adlassnig W., Lang I. (2019). Oxidative stress mediated by gyrophoric acid from the lichen Umbilicaria hirsuta affected apoptosis and stress/survival pathways in HeLa cells. BMC Complement. Altern. Med..

[42] Simko P., Leskanicova A., Suvakova-Nunhart M., Koval J., Zidekova N., Karasova M., Majerova P., Verboova L., Blicharova A., Kertys M. (2024). The First In Vivo Study Shows That Gyrophoric Acid Changes Behavior of Healthy Laboratory Rats. Int. J. Mol. Sci..

[43] Mohammadi M., Zambare V., Suntres Z., Christopher L. (2022). Isolation, characterization, and breast cancer cytotoxic activity of gyrophoric acid from the lichen Umbilicaria muhlenbergii. Processes.

[44] Sovrlic M., Manojlovic N., Kosanic M., Kocovic A., Tomović J., Vasiljević P. Lichenochemical analysis and in vitro antioxidant activity of extracts and gyrophoric acid from lichen Umbilicaria grisea. Proceedings of the 2nd International Conference on Chemo and BioInformatics.

[45] Simko P., Leskanicova A., Suvakova M., Blicharova A., Karasova M., Goga M., Kolesarova M., Bojkova B., Majerova P., Zidekova N. (2022). Biochemical Properties of Atranorin-Induced Behavioral and Systematic Changes of Laboratory Rats. Life.

[46] Urbanska N., Simko P., Leskanicova A., Karasova M., Jendzelovska Z., Jendzelovsky R., Rucova D., Kolesarova M., Goga M., Backor M. (2022). Atranorin, a Secondary Metabolite of Lichens, Exhibited Anxiolytic/Antidepressant Activity in Wistar Rats. Life.

[47] Goga M., Elečko J., Marcinčinová M., Ručová D., Bačkorová M., Bačkor M. (2020). Lichen metabolites: An overview of some secondary metabolites and their biological potential. Co-Evolution of Secondary Metabolites.

[48] Fernández-Moriano C., Gómez-Serranillos M.P., Crespo A. (2016). Antioxidant potential of lichen species and their secondary metabolites. A systematic review. Pharm. Biol..

[49] Prusinowska R., Śmigielski K.B. (2014). Composition, biological properties and therapeutic effects of lavender L). A review. Herba Pol..

[50] Shtro A., Zarubaev V., Luzina O., Sokolov D., Salakhutdinov N. (2015). Derivatives of usnic acid inhibit broad range of influenza viruses and protect mice from lethal influenza infection. Antivir. Chem. Chemother..

[51] Sokolov D.N., Zarubaev V.V., Shtro A.A., Polovinka M.P., Luzina O.A., Komarova N.I., Salakhutdinov N.F., Kiselev O.I. (2012). Anti-viral activity of (−)-and (+)-usnic acids and their derivatives against influenza virus A (H1N1) 2009. Bioorganic Med. Chem. Lett..

[52] Gupta A., Sahu N., Singh A.P., Singh V.K., Singh S.C., Upadhye V.J., Mathew A.T., Kumar R., Sinha R.P. (2022). Exploration of novel lichen compounds as inhibitors of SARS-CoV-2 Mpro: Ligand-based design, molecular dynamics, and ADMET analyses. Appl. Biochem. Biotechnol..

[53] Krishnan R., Greeshma G., Lawarence B., Murugan K. (2023). Insight of Bioresources from Lower Plant Groups: Reconciling the Possibilities and Responsibilities. Conservation and Sustainable Utilization of Bioresources.

[54] Carola V., D’Olimpio F., Brunamonti E., Mangia F., Renzi P. (2002). Evaluation of the elevated plus-maze and open-field tests for the assessment of anxiety-related behaviour in inbred mice. Behav. Brain Res..

[55] Kalin N.H. (2020). The critical relationship between anxiety and depression. Am. J. Psychiatry.

[56] Koolhaas J., Meerlo P., De Boer S., Strubbe J., Bohus B. (1995). Social stress in rats: An animal model of depression?. Acta Neuropsychiatr..

[57] Rodgers R., Cao B.-J., Dalvi A., Holmes A. (1997). Animal models of anxiety: An ethological perspective. Braz. J. Med. Biol. Res..

[58] Smith R., Taylor S., Wilson R.C., Chuning A.E., Persich M.R., Wang S., Killgore W.D. (2022). Lower levels of directed exploration and reflective thinking are associated with greater anxiety and depression. Front. Psychiatry.

[59] Salgado F., Caballero J., Vargas R., Cornejo A., Areche C. (2020). Continental and Antarctic Lichens: Isolation, identification and molecular modeling of the depside tenuiorin from the Antarctic lichen Umbilicaria antarctica as tau protein inhibitor. Nat. Prod. Res..

[60] Cazarin C.A., Dalmagro A.P., Gonçalves A.E., Boeing T., da Silva L.M., Corrêa R., Klein-Júnior L.C., Pinto B.C., Lorenzett T.S., da Costa Sobrinho T.U. (2021). Usnic acid enantiomers restore cognitive deficits and neurochemical alterations induced by Aβ1–42 in mice. Behav. Brain Res..

[61] Johnson A., Varberg Z., Benhardus J., Maahs A., Schrater P. (2012). The hippocampus and exploration: Dynamically evolving behavior and neural representations. Front. Hum. Neurosci..

[62] Pedro A., Monteiro J., Silva A.J. (2023). Perspective Chapter: Role of the Hippocampal Formation in Navigation from a Simultaneous Location and Mapping Perspective. Hippocampus-More Than Just Memory.

[63] Miller B.R., Hen R. (2015). The current state of the neurogenic theory of depression and anxiety. Curr. Opin. Neurobiol..

[64] Coe C.L., Kramer M., Czéh B., Gould E., Reeves A.J., Kirschbaum C., Fuchs E. (2003). Prenatal stress diminishes neurogenesis in the dentate gyrus of juvenile rhesus monkeys. Biol. Psychiatry.

[65] Lucassen P., Bosch O., Jousma E., Krömer S., Andrew R., Seckl J., Neumann I. (2009). Prenatal stress reduces postnatal neurogenesis in rats selectively bred for high, but not low, anxiety: Possible key role of placental 11β-hydroxysteroid dehydrogenase type 2. Eur. J. Neurosci..

[66] García-Martinez Y., Sánchez-Huerta K.B., Pacheco-Rosado J. (2020). Quantitative characterization of proliferative cells subpopulations in the hilus of the hippocampus of adult Wistar rats: An integrative study. J. Mol. Histol..

[67] Gould E., McEwen B.S., Tanapat P., Galea L.A., Fuchs E. (1997). Neurogenesis in the dentate gyrus of the adult tree shrew is regulated by psychosocial stress and NMDA receptor activation. J. Neurosci..

[68] Czéh B., Welt T., Fischer A.K., Erhardt A., Schmitt W., Müller M.B., Toschi N., Fuchs E., Keck M.E. (2002). Chronic psychosocial stress and concomitant repetitive transcranial magnetic stimulation: Effects on stress hormone levels and adult hippocampal neurogenesis. Biol. Psychiatry.

[69] Podgorny O.V., Gulyaeva N.V. (2021). Glucocorticoid-mediated mechanisms of hippocampal damage: Contribution of subgranular neurogenesis. J. Neurochem..

[70] Cameron H.A., Woolley C.S., Gould E. (1993). Adrenal steroid receptor immunoreactivity in cells born in the adult rat dentate gyrus. Brain Res..

[71] Armanini M.P., Hutchins C., Stein B.A., Sapolsky R.M. (1990). Glucocorticoid endangerment of hippocampal neurons is NMDA-receptor dependent. Brain Res..

[72] Cameron H.A., McEwen B.S., Gould E. (1995). Regulation of adult neurogenesis by excitatory input and NMDA receptor activation in the dentate gyrus. J. Neurosci..

[73] Gulyaeva N. (2021). Glucocorticoid regulation of the glutamatergic synapse: Mechanisms of stress-dependent neuroplasticity. J. Evol. Biochem. Physiol..

[74] Bartsch T. (2012). The hippocampus in neurological disease. The Clinical Neurobiology of the Hippocampus: An Integrative View.

[75] Seki T., Hori T., Miyata H., Maehara M., Namba T. (2019). Analysis of proliferating neuronal progenitors and immature neurons in the human hippocampus surgically removed from control and epileptic patients. Sci. Rep..

[76] Bond A.M., Ming G.-l., Song H. (2022). What is the relationship between hippocampal neurogenesis across different stages of the lifespan?. Front. Neurosci..

[77] Ureña-Vacas I., González-Burgos E., Divakar P.K., Gómez-Serranillos M.P. (2023). Lichen depsides and tridepsides: Progress in pharmacological approaches. J. Fungi.

[78] Reddy R.G., Veeraval L., Maitra S., Chollet-Krugler M., Tomasi S., Lohezic-Le Devehat F., Boustie J., Chakravarty S. (2016). Lichen-derived compounds show potential for central nervous system therapeutics. Phytomedicine.

[79] Porter G.A., O’Connor J.C. (2022). Brain-derived neurotrophic factor and inflammation in depression: Pathogenic partners in crime?. World J. Psychiatry.

[80] Yang T., Nie Z., Shu H., Kuang Y., Chen X., Cheng J., Yu S., Liu H. (2020). The role of BDNF on neural plasticity in depression. Front. Cell. Neurosci..

[81] Al Mamun A., Matsuzaki K., Islam R., Hossain S., Hossain M.E., Katakura M., Arai H., Shido O., Hashimoto M. (2022). Chronic administration of thymoquinone enhances adult hippocampal neurogenesis and improves memory in rats via regulating the BDNF signaling pathway. Neurochem. Res..

[82] Qin T., Yuan Z., Yu J., Fu X., Deng X., Fu Q., Ma Z., Ma S. (2020). Saikosaponin-d impedes hippocampal neurogenesis and causes cognitive deficits by inhibiting the survival of neural stem/progenitor cells via neurotrophin receptor signaling in mice. Clin. Transl. Med..

[83] Sairanen M., Lucas G., Ernfors P., Castrén M., Castrén E. (2005). Brain-derived neurotrophic factor and antidepressant drugs have different but coordinated effects on neuronal turnover, proliferation, and survival in the adult dentate gyrus. J. Neurosci..

[84] Katoh R.S., Asano T., Ueda H., Morishita R., Takeuchi I.K., Inaguma Y., Kato K. (2002). Riluzole enhances expression of brain-derived neurotrophic factor with consequent proliferation of granule precursor cells in the rat hippocampus. FASEB J..

[85] Scharfman H., Goodman J., Macleod A., Phani S., Antonelli C., Croll S. (2005). Increased neurogenesis and the ectopic granule cells after intrahippocampal BDNF infusion in adult rats. Exp. Neurol..

[86] Schmidt H.D., Duman R.S. (2010). Peripheral BDNF produces antidepressant-like effects in cellular and behavioral models. Neuropsychopharmacology.

[87] Kuipers S.D., Trentani A., Tiron A., Mao X., Kuhl D., Bramham C.R. (2016). BDNF-induced LTP is associated with rapid Arc/Arg3. 1-dependent enhancement in adult hippocampal neurogenesis. Sci. Rep..

[88] Leal G., Bramham C., Duarte C. (2017). BDNF and hippocampal synaptic plasticity. Vitam. Horm..

[89] Cardile V., Graziano A., Avola R., Piovano M., Russo A. (2017). Potential anticancer activity of lichen secondary metabolite physodic acid. Chem.-Biol. Interact..

[90] Mohammadi M., Bagheri L., Badreldin A., Fatehi P., Pakzad L., Suntres Z., van Wijnen A.J. (2022). Biological Effects of Gyrophoric Acid and Other Lichen Derived Metabolites, on Cell Proliferation, Apoptosis and Cell Signaling Pathways. Chem.-Biol. Interact..

[91] Yousuf S., Choudhary M.I. (2014). Lichens: Chemistry and biological activities. Stud. Nat. Prod. Chem..

[92] Zeb A. (2020). Concept, mechanism, and applications of phenolic antioxidants in foods. J. Food Biochem..

[93] Emsen B., Turkez H., Togar B., Aslan A. (2017). Evaluation of antioxidant and cytotoxic effects of olivetoric and physodic acid in cultured human amnion fibroblasts. Hum. Human. Exp. Toxicol..

[94] Fernández-Moriano C., Divakar P.K., Crespo A., Gómez-Serranillos M.P. (2017). In vitro neuroprotective potential of lichen metabolite fumarprotocetraric acid via intracellular redox modulation. Toxicol. Appl. Pharmacol..

[95] Fernández-Moriano C., Divakar P.K., Crespo A., Gómez-Serranillos M.P. (2017). Protective effects of lichen metabolites evernic and usnic acids against redox impairment-mediated cytotoxicity in central nervous system-like cells. Food Chem. Toxicol..

[96] Fournet M., Bonté F., Desmoulière A. (2018). Glycation damage: A possible hub for major pathophysiological disorders and aging. Aging Dis..

[97] Matés J.M., Pérez-Gómez C., De Castro I.N. (1999). Antioxidant enzymes and human diseases. Clin. Biochem..

[98] Subbaiyan R., Ganesan A., Dhanuskodi S., Prakash H.P., Varadharajan V. (2024). Virtual screening of the natural antifoulants: In silico approach to screen lichen metabolites against marine biofoulers. Environ. Prog. Sustain. Energy.

[99] Rosenbaum D.M., Rasmussen S.G., Kobilka B.K. (2009). The structure and function of G-protein-coupled receptors. Nature.

[100] Bay M.V., Nam P.C., Quang D.T., Mechler A., Hien N.K., Hoa N.T., Vo Q.V. (2020). Theoretical study on the antioxidant activity of natural depsidones. ACS Omega.

[101] Kosanic M., Rankovic B., Stanojkovic T., Vasiljevic P., Manojlovic N. (2014). Biological activities and chemical composition of lichens from Serbia. Excli J..

[102] Lobo V., Patil A., Phatak A., Chandra N. (2010). Free radicals, antioxidants and functional foods: Impact on human health. Pharmacogn. Rev..

[103] Mohamed H., Ons M., Yosra E.T., Rayda S., Neji G., Moncef N. (2009). Chemical composition and antioxidant and radical-scavenging activities of Periploca laevigata root bark extracts. J. Sci. Food Agric..

[104] Kosanić M., Ranković B., Stanojković T., Rančić A., Manojlović N. (2014). Cladonia lichens and their major metabolites as possible natural antioxidant, antimicrobial and anticancer agents. LWT-Food Sci. Technol..

[105] Melo M.G.D., dos Santos J.P.A., Serafini M.R., Caregnato F.F., de Bittencourt Pasquali M.A., Rabelo T.K., da Rocha R.F., Quintans L., de Souza Araújo A.A., da Silva F.A. (2011). Redox properties and cytoprotective actions of atranorin, a lichen secondary metabolite. Toxicol. Vitr..

[106] Siqueira R.S., Bonjardim L.R., Araújo A.A., Araújo B.E., Melo M.G., Oliveira M.G., Gelain D.P., Silva F.A., Albuquerque-Júnior R.L., Rocha R.F. (2010). Antinociceptive activity of atranorin in mice orofacial nociception tests. Z. Für Naturforschung C.

[107] Naoi M., Shamoto-Nagai M., Maruyama W. (2019). Neuroprotection of multifunctional phytochemicals as novel therapeutic strategy for neurodegenerative disorders: Antiapoptotic and antiamyloidogenic activities by modulation of cellular signal pathways. Future Neurol..

[108] Shepardson N.E., Shankar G.M., Selkoe D.J. (2011). Cholesterol level and statin use in Alzheimer disease: II. Review of human trials and recommendations. Arch. Neurol..

[109] Latkowska E., Bober B., Chrapusta E., Adamski M., Kaminski A., Bialczyk J. (2015). Secondary metabolites of the lichen *Hypogymnia physodes* (L.) Nyl. and their presence in spruce (*Picea abies* (L.) H. Karst.) bark. Phytochemistry.

[110] Bauer J., Waltenberger B., Noha S.M., Schuster D., Rollinger J.M., Boustie J., Chollet M., Stuppner H., Werz O. (2012). Discovery of depsides and depsidones from lichen as potent inhibitors of microsomal prostaglandin E2 synthase-1 using pharmacophore models. ChemMedChem.

[111] Studzińska-Sroka E., Majchrzak-Celińska A., Zalewski P., Szwajgier D., Baranowska-Wójcik E., Żarowski M., Plech T., Cielecka-Piontek J. (2021). Permeability of *Hypogymnia physodes* Extract Component—Physodic Acid through the Blood–Brain Barrier as an Important Argument for Its Anticancer and Neuroprotective Activity within the Central Nervous System. Cancers.

[112] Van den Hove D., Leibold N., Strackx E., Martinez-Claros M., Lesch K., Steinbusch H., Schruers K., Prickaerts J. (2014). Prenatal stress and subsequent exposure to chronic mild stress in rats; interdependent effects on emotional behavior and the serotonergic system. Eur. Neuropsychopharmacol..

[113] Gerdes J., Lemke H., Baisch H., Wacker H.-H., Schwab U., Stein H. (1984). Cell cycle analysis of a cell proliferation-associated human nuclear antigen defined by the monoclonal antibody Ki-67. J. Immunol..

[114] Kee N., Sivalingam S., Boonstra R., Wojtowicz J. (2002). The utility of Ki-67 and BrdU as proliferative markers of adult neurogenesis. J. Neurosci. Methods.

[115] Leskanicova A., Babincak M., Mochnacky F., Pipova Kokosova N., Kukelova D., Urbanska N., Kolesarova M., Macekova D., Kostolny J., Kiskova T. (2021). Sex-dependent differences in stress-induced depression in Wistar rats are accompanied predominantly by changes in phosphatidylcholines and sphingomyelins. J. Physiol. Pharmacol..

[116] Pipová Kokošová N., Kisková T., Vilhanová K., Štafuriková A., Jendželovský R., Račeková E., Šmajda B. (2020). Melatonin mitigates hippocampal and cognitive impairments caused by prenatal irradiation. Eur. J. Neurosci..

